# Modulation of spontaneous alpha brain rhythms using low-intensity transcranial direct-current stimulation

**DOI:** 10.3389/fnhum.2013.00529

**Published:** 2013-09-03

**Authors:** Grazia F. Spitoni, Rocco L. Cimmino, Chiara Bozzacchi, Luigi Pizzamiglio, Francesco Di Russo

**Affiliations:** ^1^Department of Psychology, Spienza University of RomeRome, Italy; ^2^Neuropsychology Unit, IRCCS Santa Lucia FoundationRome, Italy; ^3^Department of Human Movement, Social and Health Sciences, University of RomeForo Italico, Italy

**Keywords:** tDCS-EEG, parietal cortex, alpha rhythm, monocephalic montage, noninvasive electric stimulation

## Abstract

Transcranial direct-current stimulation (tDCS) is a form of neurostimulation in which a constant, low current is delivered directly to the brain area of interest by small electrodes. The overall aim of this study was to examine and monitor the modulation of brain activity by electroencephalogram (EEG) in the frequency domain during tDCS in the resting state. To this end, we considered the modulation of spontaneous EEG to be a marker of the perturbation that was induced through the direct current (1.5 mA for 15 min). In all conditions (anodal, cathodal, and sham), an active electrode was placed over the right posterior parietal cortex, and a reference electrode was placed on the ipsilateral deltoid muscle. The EEG was recorded using a 64-channel system. The effect of tDCS was limited to the alpha rhythm, and the anodal stimulation significantly affected the alpha rhythm, whereas the cathodal stimulation did not elicit any modifications. Further, we observed modulation of alpha activity in areas that were stimulated directly through tDCS and in anterior noncontiguous areas. Finally, the anodal effect peaked 7.5 min after stimulation and decreased gradually over time. Our study demonstrates that in the resting brain, monocephalic anodal tDCS over posterior parietal areas alters ongoing brain activity, specifically in the alpha band rhythm. Our data can be used to fine-tune tDCS protocols in neurorehabilitation settings.

## Introduction

Transcranial direct-current stimulation (tDCS) is a noninvasive technique that modulates the neuronal excitability of targeted cerebral areas through constant stimulation with low direct current (DC) from the scalp using a pair of electrodes. Physiological studies have demonstrated that DC flows through the skull and the outer layers of the cortex, modifies neuronal crossmembrane resting potentials, influences the level of neuronal excitability, and modulates firing rates (Nitsche et al., 2011). Depending on the orientation of the cells with respect to the current, the membrane potentials can be hyperpolarized (anodal stimulation) or depolarized (cathodal stimulation) by several mV (Paulus, 2004). This change in neuronal excitability effects several alterations in brain function (Nitsche et al., 2008), including motor, sensory, and high-level cognitive function (Calvo-Merino and Haggard, 2004; Nitsche et al., 2005).

Modulation of behavior through noninvasive brain stimulation to enhance or reduce performance is a valuable tool for research and rehabilitation. Since the publication of seminal studies on motor tasks (Rosenkranz et al., 2000; Lang et al., 2004), this discipline has focused increasingly on the effects of tDCS on various cognitive domains, such as language (Floel et al., 2008; Fertonani et al., 2010; Fiori et al., 2011), spatial attention (Bolognini et al., 2010), executive functions (Dockery et al., 2009; Hecht et al., 2010), visual processing (Antal and Paulus, 2008), body representation (Spitoni et al., 2013), and emotions (Boggio et al., 2008), and on its implications for neuropsychological rehabilitation (Vallar and Bolognini, 2011).

Several electrical stimulation techniques have been used in experimental contexts and rehabilitative settings. Electrical stimulation methods, such as tDCS, transcranial alternating current stimulation (tACS), and transcranial random noise stimulation (tRNS), alter spontaneous cortical activity (Kuo and Nitsche, 2012).

Studies have examined EEG oscillations following tDCS, but most have centered around its effects on motor and cognitive tasks. For example, Ardolino et al. (2005) reported that cathodal stimulation of the motor cortex increases the power of delta and theta rhythms, whereas Polania et al. (2011) demonstrated that after anodal stimulation over the primary motor cortex (M1), functional connectivity patterns increased significantly in the premotor, motor, and sensory motor areas of the stimulated hemisphere during motor activity. Electrophysiological changes were also observed following stimulation over nonmotor areas.

On a working memory (WM) task (*n*-back), Keeser et al. (2011) showed that 20 min of anodal DC (2 mA) over the left dorsolateral prefrontal cortex (DLPFC) significantly reduced left frontal delta activity. Further, Zaehle et al. (2011) stimulated the left DLPFC during a WM task and reported a significant reduction in mean current densities in the delta band following anodal stimulation and changes in theta and alpha band activities. Finally, in a study on motor imagery, Matsumoto et al. (2010) noted that Mu event-related desynchronization rose significantly after anodal stimulation of M1 and declined after cathodal stimulation. Thus, these studies document the efficacy of weak currents in modulating neuronal excitability and behavioral performance on cognitive and motor tasks.

The field of noninvasive brain stimulation has attracted increasing interest with regard to ongoing oscillatory brain activity during rest, which potentially constitutes an index of the internal state of the brain in the absence of an external input or motor output (Markman and Dietrich, 2000); yet, few studies (e.g., Ardolino et al., 2005) have explicitly investigated the effects of tDCS on oscillatory brain activity at rest. In this study, we examined the effects of tDCS on spontaneous cortical activity to determine the modulation of spontaneous oscillatory brain activity in a resting brain that has been perturbed electrically through anodal and cathodal stimulation. To this end, we measured the modulation of spontaneous EEG to describe the alterations that are induced through tDCS.

We also wished to determine the duration of the effects of tDCS. Several minutes of stimulation induces aftereffects that persist from minutes to hours (Paulus, 2004). For example, Antal and Paulus (2008) reported that DC stimulation had a significant effect between 5 and 10 min after anodal stimulation in the V5motion visual area and from 10 to 15 min after anodal and cathodal tDCS over the primary motor area. These effects remained stable for 25 min and diminished gradually after several hours. Further, Keeser et al. (2011) demonstrated that the effect of tDCS was stronger in the first 5 min of stimulation. In contrast, no systematic study has examined the duration of the effect of tDCS over time in a resting state—data that have implications regarding tDCS as rehabilitation therapy.

In tDCS, the electrode placement varies, depending on the nature of the experimental hypothesis. Two montages are typically used: bicephalic and monocephalic. Nitsche et al. (2011) suggested that the monocephalic montage avoids the confounding effects of the reference electrode. Thus, we used a monocephalic montage with an electrode placed over the posterior parietal areas and another electrode on the right shoulder (Da Silva et al., 2011). Although this configuration modulates the neuronal excitability of the brainstem, we hypothesized that the electrical effect of DC would primarily affect the cortex under and around the active electrode and reduce the effects in areas that were distal to the active electrode (Miranda et al., 2006; Wagner et al., 2007).

We stimulated the posterior parietal lobe for 2 overarching reasons: no EEG study has specifically examined the effects of tDCS in this area, and tDCS has been used as a rehabilitation tool for visuospatial deficits. Thus, we wanted to describe the modulation of activity in this area pre- and poststimulation.

Our aim was to investigate the electrophysiological changes that are induced through anodal and cathodal tDCS over posterior parietal areas during the resting state. The practical implication relies on the possibility to provide further support for fine-tuning rehabilitative tDCS protocols in treating patients who suffer from deficits in visuospatial attention domains.

## Materials and methods

### Participants

Nineteen subjects participated in this study. Four subjects were excluded due to a significant amount of muscular artifacts on the EEG. The remaining 15 participants (8 females) were right-handed, as assessed using a modified version of the Edinburgh Inventory (mean handedness 95 ± 12) (Salmaso and Longoni, 1985). The participants ranged in age from 21 to 34 years (mean age 23.3; *SD* = 3.4).

The inclusion criteria were: (1) no history of neurological or psychiatric disorders; (2) no history of substance abuse or dependence; and (3) no use of medication that affected the central nervous system. All participants provided written, informed consent per the Code of Ethics of the World Medical Association (Declaration of Helsinki). The study was approved by the ethics committee of IRCCS Santa Lucia Foundation, Rome.

### tDCS stimulation

We used the safety protocol of Brunoni et al. (2012). Briefly, a DC of 1.5 mA (impedance limit, 50 kOhm), induced through 2 saline-soaked surface sponge electrodes (7 × 4.5 cm), was delivered using a battery-driven, constant-current DC stimulator (neuroConn GmbH, Ehrenbergstr, Ilmenau, Germany).

To avoid confounding biases that could have arisen from 2 electrodes with opposite polarities over the scalp, we used a noncephalic reference electrode for tDCS (Cogiamanian et al., 2007; Priori et al., 2008). Under both anodal and cathodal conditions, the active electrode was placed over the right posterior dorsal parietal lobule, and the reference electrode was placed over the ipsilateral deltoid muscle. The location of the active electrode was determined per the 10-10 EEG standard montage, placing the electrode over P2, P4, and P6, as suggested in previous studies. The strongest effect of tDCS is observed under and around the active electrode (Okamoto et al., 2004; Fuggetta et al., 2006; Miranda et al., 2006; Wagner et al., 2007). Thus, the site of stimulation that we chose likely affected the right angular gyrus.

In the stimulation sessions, the current was ramped from 0 to 1.5 mA in 60 s. Onset of stimulation elicited a transient tingling sensation on the scalp (Hummel and Cohen, 2005). Fifteen minutes after onset, the current was turned off slowly over 60 s. In the sham condition, the electrodes were placed in the same positions as in the anodal/cathodal conditions, but the device was decreased gradually after 60 s (30 s ramp up and 30 s ramp down). This procedure ensured that the participants felt the typical tingling sensation at the beginning of the stimulation (Gandiga et al., 2006).

### Procedure

Participants were seated in a quiet room and asked verbally every 30 s to open or close their eyes. During the open-eye period, the subjects were instructed not to move their eyes from a fixed point in front of them. Table 1 shows the stimulation and recording protocols. To avoid carryover effects, the experimental sessions were separated by at least 5 days. The participants did not know whether actual tDCS (anodic or cathodic) or sham simulation was administered.

**Table 1:** **Sequence of stimulation and recording**.

	**EEG recording pretest**	**tDCS**	**EEG recording sham**	**tDCS**	**EEG recording posttest**
Session 1	15 min	15 min sham	15 min	15 min anodal	15 min
Session 2	15 min	15 min sham	15 min	15 min cathodal	15 min

During spontaneous EEG recording, the eyes were opened or closed every 30 s.

After the first 15 min of EEG recordings (pretest), the electrode cap was disconnected from the amplifiers, and the tDCS electrode was inserted under the cap beneath P2, P4, and P6. The time that was needed to place the tDCS electrode ranged from 1 to 2 min. The sham stimulation was administered for 15 min. Subsequently, the tDCS electrode was removed, and the impedance of the 64 electrodes was measured, particularly those at P2, P4, and P6. The EEG recording lasted 15 min, after the absence of artifacts was verified. The same electrode placement method was repeated for the anodic/cathodic stimulation. The time from the end of stimulation to the recording of data from all 64 channels was 2 min.

### EEG recording and analysis

The EEG was recorded using a BrainVision system from 64 electrodes that were placed per the 10-10-system montage (Di Russo and Spinelli, 2002). Tin electrodes (instead of silver) were used to avoid the polarization that is induced by tDCS. All channels were initially referenced to the left mastoid (M1), and the ground electrode was located to the CPz. Horizontal eye movements were monitored by bipolar recording from electrodes at the right corner of the eyelid. Blinks and vertical eye movements were recorded by an electrode under the left eye, which was referenced to the Fp1.

The impedance of the electrodes was monitored periodically and maintained below 10 kOhm throughout the experiment. The EEG from each electrode site was digitized at 250 Hz using an amplifier bandpass of 0.01–80 Hz, including a 50-Hz notch filter, and stored for offline averaging. Under open- and closed-eye conditions, the EEG data were segmented into single 30-s epochs, adjusted through ocular correction (Gratton et al., 1983), and filtered (2–50 Hz). Computerized artifacts were rejected to discard segments in which deviations in eye movements, blinking, and physical artifacts occurred (difference criterion 100 μV). Thus, only EEG segments that were free of artifacts were accepted for fast Fourier transformation (FFT) using a resolution of 0.5 Hz and a Hanning window of 10% of the length. The results were expressed power values (μ V^2^).

In a preliminary analysis, the entire EEG spectrum was analyzed and divided into the 5 chief frequency bands: 2–4 Hz (delta), 4–8 Hz (theta), 8–12 Hz (alpha), 13–30 Hz (beta), and 30–50 Hz (gamma). The averaged power of each frequency band of the peak channel in the stimulated area (P2, P4, and P6) was used for the analysis. By five 2 × 2 repeated measures ANOVAs, with test (pretest = 15 min and posttest = 15 min) and eyes (open or closed) as factors, we noted a significant effect of anodal stimulation in the alpha band (see Table 2). Thus, these analyses focused solely on alpha activity.

**Table 2:** **Effects of stimulation by frequency band**.

**Frequency band**	***F*_(1, 14)_ (anodal)**	**Significance**	***F*_(1, 14)_ (cathodal)**	**Significance**
Delta	0.36	0.73	0.45	0.62
Theta	0.22	0.64	0.58	0.45
Alpha	6.03	0.02	0.29	0.59
Beta	0.35	0.56	4.06	0.06
Gamma	0.49	0.49	0.17	0.68

**Figure 1:**
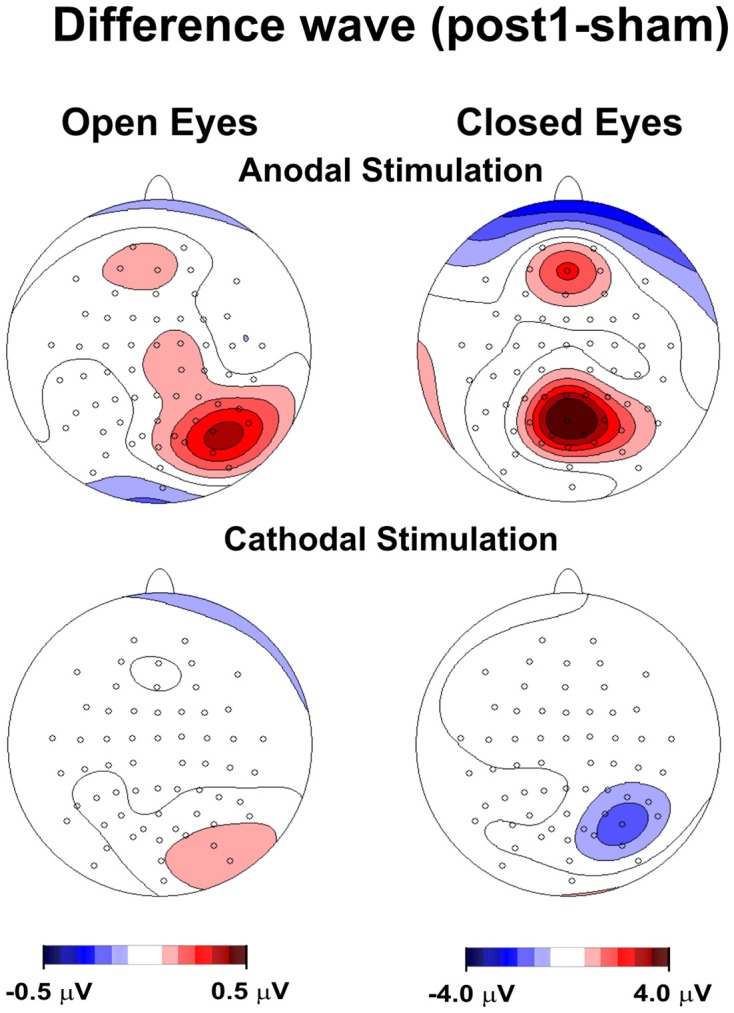
**Topography of the effect of tDCS for open and closed eyes and anodal and cathodal stimulation**. The maps were obtained by subtracting the sham condition from posttest1.

The averaged power of the alpha frequency band was calculated for each participant and used for the statistical analysis of 3 areas of interest, based on the topography of the alpha—1 peak each over midline occipitoparietal sites, right parietal sites, and medial frontal sites. The medial occipital region was defined as Pz, POz, and Oz (where alpha activity is usually prominent); the right parietal region was defined as P2, P4, and P6 (corresponding to the stimulated area); and the medial frontal region was defined as AFz, Fz, and FCz (Figure 1).

To examine changes in the effects of tDCS, the posttest period was divided into 2 segments: posttest1 (the first 7.5 min) and posttest2 (the subsequent 7.5 min).

Alpha power scores were analyzed by 2 × 2 × 4 within-subjects repeated measures ANOVA, with stimulation (anodal and cathodal), eyes (open or closed), and time (pretest, sham, posttest1, and posttest2) as factors. The analyses were performed for each area of interest in the parietal, occipital, and frontal regions. Bonferroni *post-hoc* tests (*p* < 0.05) were also conducted.

Further, the effects of tDCS over time were analyzed by 9 × 2 within-subjects repeated measures ANOVA, with time (9 levels) and eyes (open or closed) as factors. The 9 levels of the factor “time” were the pretest and 8 epochs of approximately 2 min (110 s) each in the posttest. As in the previous analysis, Bonferroni *post-hoc* tests (*p* < 0.05) were performed. The topography of the scalp was also examined using spline-interpolated maps, focusing on the effects of stimulation on the alpha rhythm. To this end, waves of the difference between posttest1 and sham were generated.

## Results

### EEG rhythm and topography

Figure 1 shows the topography of posttest1-minus-sham activity, in which the frontoparietal alpha band modulated following anodal, but not cathodal, tDCS in the closed-eye condition. We also observed a drift toward a more medial distribution.

**Figure 2:**
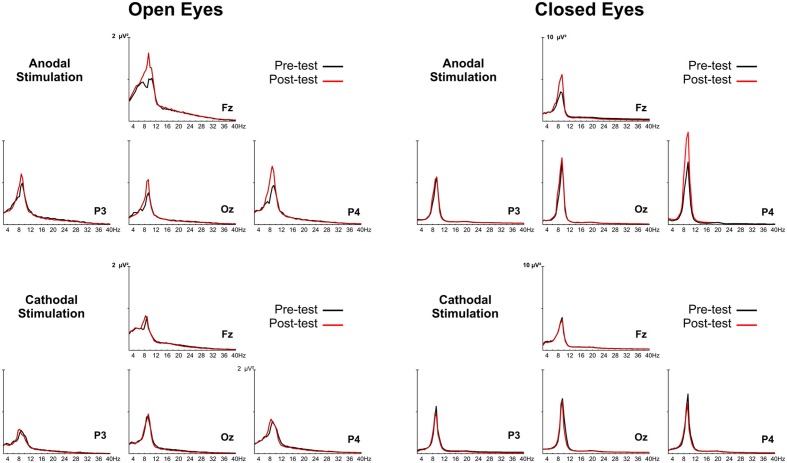
**Group grand-averaged EEG spectra of anodal and cathodal stimulation with the eyes open (left panel) and closed (right panel)**. The black line represents activity before tDCS (pretest), and the red line is activity after tDCS (posttest).

The group-averaged power spectra of the EEG during anodal and cathodal stimulation sessions for open- and closed-eye conditions are reported in Figure 2.

In the parietal region, by ANOVA, there was a main effect of stimulation [*F*_(1, 14)_ = 8.56, *p* < 0.01], eyes [*F*_(1, 14)_ = 20.05, *p* < 0.00], and time [*F*_(3, 42)_ = 6.39, *p* < 0.00], indicating that there were substantial differences between the levels of the 3 factors. Moreover, the significant interaction “stimulation × eyes × time” [*F*_(3, 42)_ = 2.66, *p* = 0.03] suggested that stimulation was differentially impacted, based on the intervening levels of time and eye.

Specifically, by *post-hoc* tests, pretest and posttest1 conditions differed significantly (*p* < 0.00), but pretest did not differ from sham or posttest2 (*p* = 1.00 and *p* = 1.00, respectively). This effect was achieved only in the anodal condition, when the eyes were closed. The remaining *post-hoc* comparisons revealed a significant difference between sham and posttest1 (*p* < 0.001) and between posttest1 and posttest2 (*p* < 0.01). No difference was observed between sham and posttest2 (*p* = 0.92).

Similarly, in the frontal region, by ANOVA, we observed a main effect of stimulation [*F*_(1, 14)_ = 12.4, *p* < 0.003], eyes [*F*_(1, 14)_ = 41.86.05, *p* < 0.001], and time [*F*_(3, 42)_ = 9.34, *p* < 0.001]. These effects indicated that there were significant differences between the levels of the 3 factors. Moreover, the significant interaction “stimulation × eyes × time” [*F*_(3, 42)_ = 6.87, *p* < 0.001] suggested that stimulation was differentially impacted, based on the intervening levels of time and eye.

Specifically, the *post-hoc* tests revealed a significant difference between pretest and posttest1 conditions (*p* < 0.001) but not between pretest and sham or posttest2 (*p* = 1.00 and *p* = 1.00, respectively). This effect was achieved only in the anodal condition with closed eyes. The remaining *post-hoc* comparisons showed a significant difference between sham and posttest1 (*p* < 0.001) and between posttest1 and posttest2 (*p* < 0.01). No difference was observed between sham and posttest2 (*p* = 1.00).

In the occipital region, by ANOVA, we observed a main effect of stimulation [*F*_(1, 14)_ = 7.43, *p* < 0.01] and eye [*F*_(1, 14)_ = 23.37, *p* < 0.001] but not time [*F*_(1, 14)_ = 1.08, *p* = 0.366]. These data indicate that there was no effect of stimulation on time in the posterior area. No other significant effects were observed [stim × eyes × time interaction: *F*_(3, 42)_ = 0.96, *p* = 0.41].

**Figure 3:**
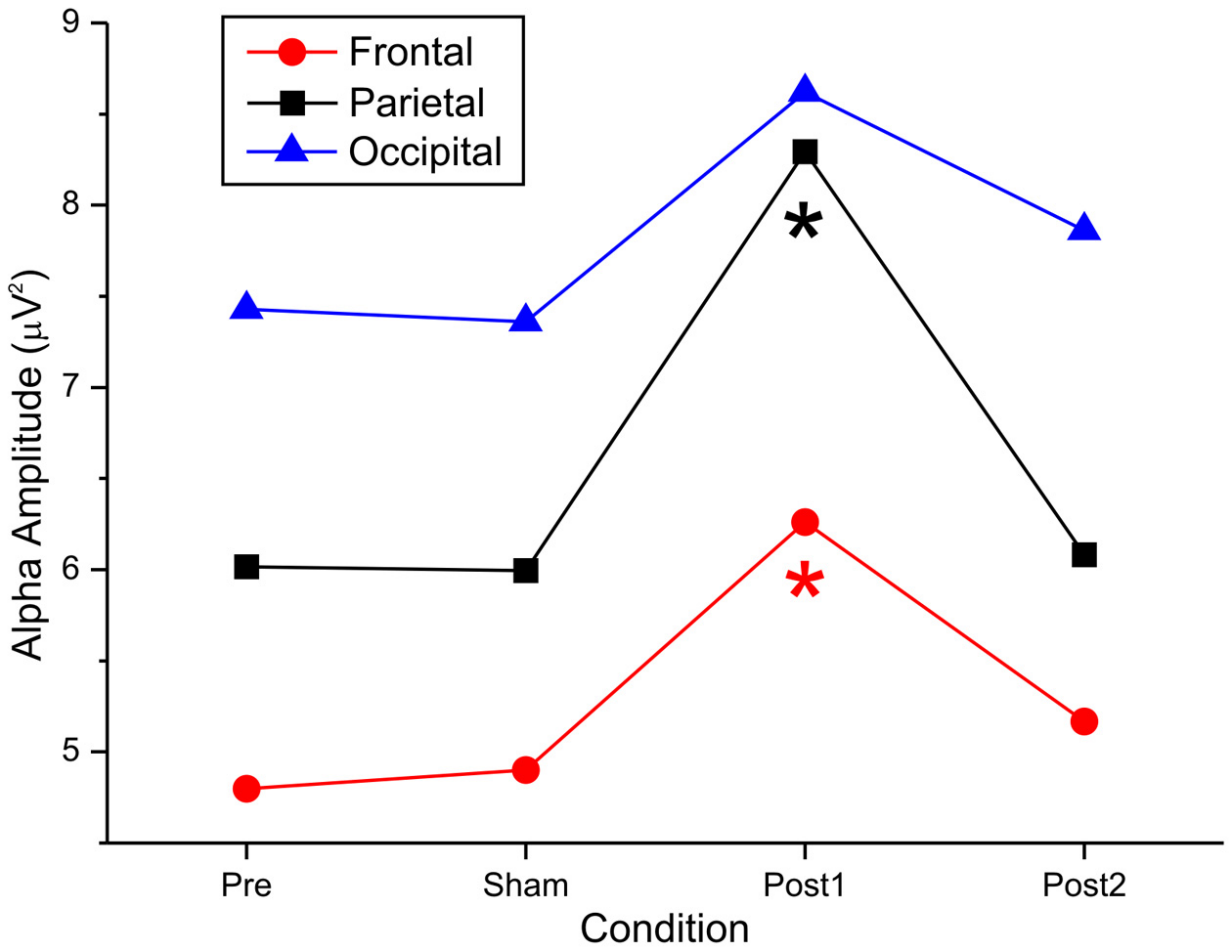
**Effect of anodal tDCS on the alpha amplitude recorded from the frontal, parietal, and occipital electrodes**.

Figure 3 summarizes the principal findings of the *post-hoc* comparisons in the 3 areas of interest and shows the statistical comparisons. Alpha power was enhanced after anodal tDCS but remained stable after cathodal stimulation; this effect existed in distant cortical regions, such as the frontal area.

**Figure 4:**
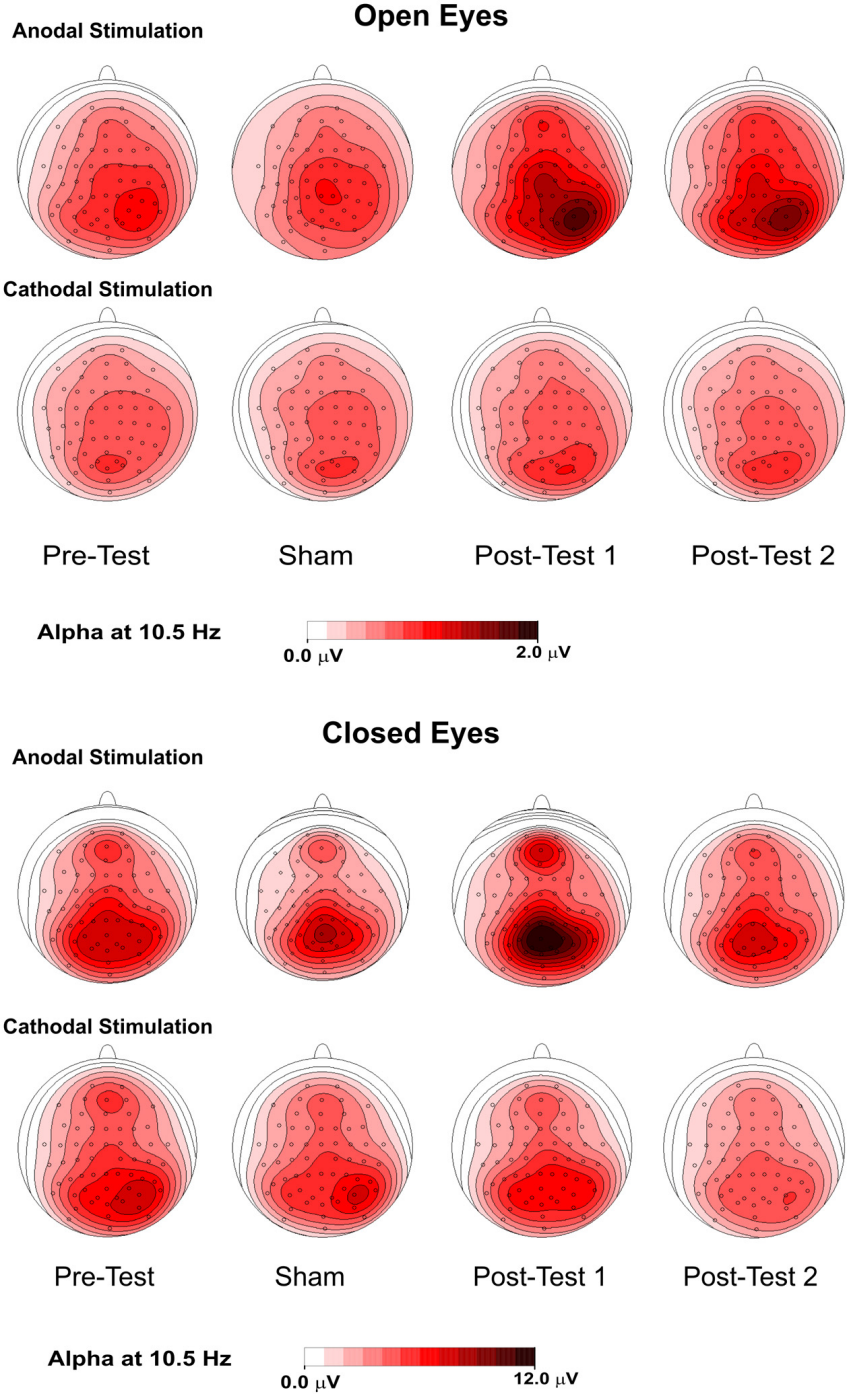
**Topographical maps (top flat view) of the alpha rhythm at 10.5 Hz in 4 conditions—pretest, sham, posttest1, and posttest2—with the eyes were open (upper panel) and closed (lower panel)**.

Figure 4 shows the topographical maps of the alpha rhythm peak (10.5 Hz) for open- and closed- eye conditions. Despite the lack of a significant effect in the open-eye condition, there was less medial and prominent alpha activity in the right parietal area. With both open and closed eyes, alpha power in the pretest and sham conditions was similar and most prominent in medial bilateral posterior parietooccipital electrodes. Notably, lower but consistent activity was also present in the medial frontal electrodes. Further, under both eye conditions, spontaneous alpha activity was higher in posttest1 and lower in posttest2 during anodal stimulation. For the open-eye period, the topography in the posttests was less medial and more prominent in the right parietal areas. This effect was also present in the closed-eye condition but less pronounced.

### tDCS effect over time

**Figure 5:**
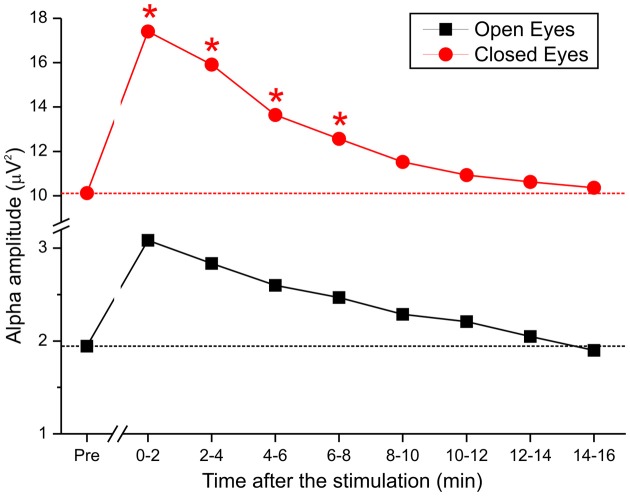
**Time course of alpha activity during pretest and after the end of anodal tDCS in 8 epochs of 2 min each**.

As described, we examined the effects of tDCS over 15 min. Based on the lack of a cathodal effect, we focused on anodal stimulation. To monitor the effect of stimulation over time in greater detail, the poststimulation recording was divided into 8 epochs of approximately 2 min each, and the alpha amplitudes of the pretest were compared with those of the 8 epochs (Figure 5). By repeated measures ANOVA, there was a significant main effect of anodal stimulation [*F*_(8, 112)_ = 2.92, *p* < 0.05] and a significant interaction between stimulation and eye [*F*_(8, 112)_ = 2.27, *p* < 0.02].

These data demonstrate that when the eyes were closed, DC was effective for approximately 8 min but lacked efficacy when the eyes were open. Specifically, by *post-hoc* test, the difference between pretest and stimulation epochs was maintained over the first 4 epochs (t1 *p* < 0.02; t2 *p* < 0.04; t3 *p* < 0.01; t4 *p* < 0.01), after which the effect was insignificant.

## Discussion

In this study, we applied anodal and cathodal tDCS over the right dorsal posterior parietal areas to examine the effects of stimulation on spontaneous EEG rhythms. Four main findings emerged: (1) the tDCS effect was limited to the alpha rhythm band; (2) anodal tDCS affected the alpha rhythm, but cathodal tDCS did not; (3) the alpha activity was modulated in noncontiguous frontal areas, but stronger modulation was observed in the parietal areas under the active stimulating electrode; and (4) the anodal effect was greater at the beginning of the period following stimulation (i.e., 7.5 min after tDCS ended) and diminished over time.

### Selective effect of anodal tDCS on alpha band rhythm

Anodal stimulation has effects only on the alpha rhythm, which was the predominant activity, because large alpha amplitudes reflect a brain state with decreased information processing (Pfurtscheller, 2001).

The selective effect of anodal stimulation requires further consideration. The common assumption that the anode electrode enhances cortical excitability whereas the cathode electrode has the opposite effect was examined recently in a meta-analysis of the polarity effects of tDCS in motor and cognitive tasks (Jacobson et al., 2012). The chief finding was that anodal and cathodal stimulation generally has the opposite effect (increasing and decreasing excitability, respectively) when applied over motor regions to influence motor functions.

However, anodal stimulation and cathodal stimulation have disparate effects on cognitive functions. Specifically, Jacobson et al. demonstrated that the likelihood of generating significant anodal effects in the cognitive domain and nonmotor areas always exceeded that of producing cathodal effects, suggesting that anodal effects are considerably more robust and reproducible than cathodal effects.

This group proffered several explanations for the lack of consistent results of the cathodal electrode over nonmotor areas. The more persuasive explanation is based on the state of the brain before stimulation. Silvanto et al. (2008) suggested that the effects of brain stimulation are dictated by the initial state of neuronal activation. During cognitive demand, the areas of the brain that support the tasks (i.e., executive, spatial, and attention tasks) become highly activated through cognitive requests during stimulation, whereas motor areas are less active during stimulation, particularly when no voluntary activation is required. Consequently, the effects of stimulation persist in a low-competition environment and can be fully expressed.

The increase in alpha amplitude is commonly associated with cortical deactivation and inhibition (Klimesch et al., 2007). Because anodal tDCS is typically linked to greater cortical excitability, a decline in alpha amplitude after anodal stimulation is expected. However, our data showed the opposite result, perhaps because such a phenomenon reflects specific effects of tDCS on inhibitory neurons, which could increase the alpha amplitude.

However, only 15% of cortical neurons are inhibitory; the remaining 85% are excitatory (Braitenberg and Schulz, 1991). Large alpha amplitudes might stem from high synchrony between a few neurons, with the majority of neurons relatively inactive (Nunez and Silberstein, 2000). Thus, we speculate that anodal stimulation excites inhibitory cells in the parietal cortex.

Alternatively, in a state of relaxed, but alert, wakefulness (i.e., participants who were required to open and close their eyes), pronounced alpha activity is observed (Klimesch, 1999). It has been suggested that after a cognitive or behavioral response (such as “opening” or “closing” the eyes), the subject relaxes and waits for the next command. In studies on the effects of anodal stimulation on various components of attention, tDCS enhances attentive functions. Based on this evidence, we propose that the increased alpha amplitude reflects enhancement of the relaxed/alert wakefulness state.

Moreover, several TMS studies (Fuggetta et al., 2005, 2008; Brignani et al., 2008) have reported widespread synchronization of alpha and beta activity after low-frequency Repetitive transcranial magnetic stimulation (rTMS) over M1. Veniero et al. (2011) demonstrated that high-frequency rTMS over motor cortex modulates the spontaneous ongoing EEG, resulting in synchronization in the alpha and beta frequency bands. Further, alpha synchronization increased as a function of the number of stimuli that were delivered, and the induction outlasted the end of TMS by over 5 min. Based on previous conclusions (Paus et al., 2001; Rosanova et al., 2009), this group interpreted these effects as the ability of an external perturbation to rearrange the ongoing oscillatory activity and unmask intrinsic oscillations and produce a commune cycle.

### Effects of tDCS of the frontoparietal network

Notably, there was significant alpha modulation in the frontal brain regions, far from the stimulated parietal site. This frontal effect was observed in nearly all subjects in the resting state with their eyes closed (13 subjects) and open (11 subjects). These data support previous findings on the “functional coupling of alpha” (Sauseng et al., 2005).

Alpha modulation is observed not only in typical posterior areas but also over more anterior synchronized regions. Klimesch et al. (2007) described this occurrence using the concept of “traveling alpha waves,” suggesting that the phase analysis of ongoing oscillations between recording sites has potentiated the detection of “traveling” alpha waves between anterior and posterior sites.

Frontoparietal alpha coupling during resting states has been examined. Laufs et al. (2003) reported that the spontaneous fluctuations in alpha oscillation power correlated negatively with activity in the dorsal attention system of the superior frontal and intraparietal regions. Mantini et al. (2007) obtained similar results in an EEG/fMRI study on resting state networks in the human brain. Sadaghiani et al. (2012) combined EEG and fMRI during resting wakefulness and showed that fluctuations in global synchrony in the upper alpha band were linked to activity in several prefrontal and parietal regions. In an analysis of fMRI intrinsic connectivity, the group confirmed that these regions correspond to the well-known frontoparietal network and suggested that this selective and specific relationship reflects an intrinsic association of large-scale alpha phase locking with cognitive operations supported by this network.

Frontoparietal alpha coupling at rest has also been examined clinically, particularly in pathological aging. Abnormalities in EEG rhythms in dementia are associated with altered regional cerebral blood flow (rCBF)/metabolism and cognitive function. In a study on mild cognitive impairments (MCIs) in aging, Babiloni et al. (2006) demonstrated that at the group level, frontoparietal coupling of delta and alpha rhythms becomes progressively abnormal in MCIs and mild Alzheimer disease, suggesting that an EEG-based approach can help predict cognitive decline in individuals who suffer from MCIs.

With regard to frontoparietal alpha coupling during cognitive demands, Sauseng et al. (2005) reported that on a WM task, alpha power increases in the prefrontal regions but declines in the occipital electrodes, wherein alpha synchronization is stronger at prefrontal sites and occipital alpha suppression is greater when the WM demand is higher. Further, Schack and Weiss (2005) examined alpha phase synchronization during the encoding and memorization of spoken words, observing a pattern of stable phase relations primarily between leading parietotemporal and trailing anterior sites, indicating that activation occurs from the posterior to anterior regions of the brain. This pattern was active for concrete but not abstract words. A robust link between alpha activity at anterior and posterior sites reflects the time at which cerebral regions are coactivated during interactive top-down and bottom-up processing.

These studies support a model in which the frontoparietal network is activated, depending on the type of task, and traveling alpha waves reflect the spread of cortical activation—i.e., 1 region of the brain controls activation in another region in a top-down manner. Our data demonstrate that anodal tDCS modulates the alpha activity of the entire network, even if the effective electrode lies only in posterior regions.

tDCS of parietal areas is used typically in the examination and rehabilitation of visuospatial deficits, such as neglect and neglect-like symptoms (for an exhaustive review, see Hesse et al., 2011). In existing studies, anodal and cathodal electrodes were positioned in parietal sites; thus, our data provide further insights into the use of tDCS to stimulate parietal regions.

### Time course of tDCS effects

The effect of tDCS over time is a critical issue, because the aftereffects of stimulation might last minutes to hours, depending on the intensity and time of exposure of the stimulation. Nitsche and Paulus (2000) suggested that at least 3 min of exposure at an intensity of at least 0.6 mA is required to obtain consistent aftereffects. According to a recent study, many factors influence the interval of the effects of tDCS. For example, Antal et al. (2010) found that at equal intensity and duration, the effects of stimulation lasted longer on motor areas than posterior regions.

Recently, Paulus (2011) reviewed the literature on the technical features of tDCS and tACS and noted a direct relationship between the duration of stimulation and the aftereffect. In our study, the strongest change occurred in the first 2 min after the stimulation ended. The effect diminished systematically and was effective for approximately 8 min, suggesting that tDCS affects EEGs immediately after stimulation.

### Limits

The methodological limits of our study should be considered. In an ideal experimental design, all conditions must be counterbalanced; however, in our study, the sham experiments always preceded active tDCS. Because alpha is expected to rise over time and with fatigue, it is unknown whether the climb in alpha after anodal tDCS is an actual effect of stimulation or simply a physiological increase.

This apparent methodological limitation can be overcome if we consider the following points. The first point concerns the intrinsic constraints of tDCS proper. Unfortunately, in tDCS studies, it is difficult to balance sham and stimulation in the same session, due to the aftereffects of tDCS. Specifically, if anodal/cathodal stimulation precedes sham stimulation, the latter can be altered by the earlier step. Thus, we were obligated to consistently perform sham stimulation before tDCS.

Further, even without counterbalancing, our data explain the nature of the effect of tDCS on alpha rhythms. Primarily, for cathodal stimulation, alpha activity increased only in the posttest1 condition (approximately 7.5 min after stimulation) and declined in the posttest2 condition (from 7.5 to 15 min after stimulation), and after cathodal stimulation, the alpha did not change. If alpha had risen over time independently from stimulation, we should have observed increased activity after both anodal and cathodal stimulation in the posttest2 condition. No tasks were requested of the subjects; thus, we did not expect a fatigue-related increase in alpha.

## Conclusion

In this study, we have demonstrated that in a resting brain, monocephalic anodal tDCS over posterior parietal areas alters ongoing brain activity, specifically in the alpha band rhythm; this effect was significant until approximately 8 min after the stimulation. Although further studies are needed to determine the optimal stimulation parameters for longlasting and efficient modulation in therapeutic applications, our data contribute to the fine-tuning of rehabilitative tDCS protocols.

### Conflict of interest statement

The authors declare that the research was conducted in the absence of any commercial or financial relationships that could be construed as a potential conflict of interest.
